# Supplementary material for: Derivation and validation of a non-invasive optoacoustic imaging biomarker for detection of patients with intermittent claudication
Source: Commun Med (Lond). 2025 Mar 25;5:88. doi: 10.1038/s43856-025-00801-1 (PMC11937270; doi:10.1038/s43856-025-00801-1)
Supplement: Supplementary file 1 — Supplementary Information [file 43856_2025_801_MOESM1_ESM.pdf]

Supplementary Information

Supplement to: Caranovic et al., Derivation and validation of a non-invasive optoacoustic imaging biomarker for detection of patients with intermittent claudication

This supplementary appendix has been provided to give readers additional information about the work.

Table of Contents

Supplementary Figures 3

Supplementary Figure 1 – VASCUQOL-6 questionnaire .... **Error! Bookmark not defined.**

Supplementary Figure 2 – TASC II classification: Aorto-iliac segment (AI) ..... **Error! Bookmark not defined.**

Supplementary Figure 3 – TASC II classification: Femoral popliteal segment (FP) .... **Error! Bookmark not defined.**

Supplementary Figure 4 – TASC II classification: Infrapopliteal segment (IF) **Error! Bookmark not defined.**

Supplementary Figure 5 – Schematic illustration of the heel raise exercise 4

Supplementary Figure 6 – Correlation of blood hemoglobin level and MOST-derived hemoglobin parameters 5

Supplementary Figure 7 – Gender differences in MSOT-derived parameters 6

Supplementary Tables 7

Supplementary Table 1 – aTASC classification based on the TASC II classification 7

Supplementary Table 2 – Demographic and Clinical Characteristics 9

Supplementary Table 3 – Representation and validation of the diagnostic quality of the MSOT measurement 10

References 11

Supplementary Figures

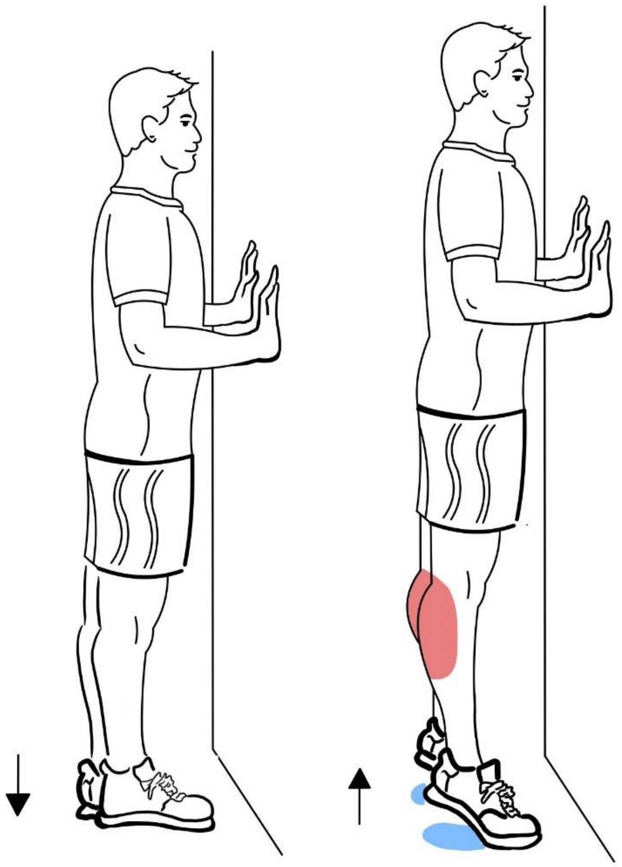

Supplementary Figure 1 – Schematic illustration of the heel raise exercise

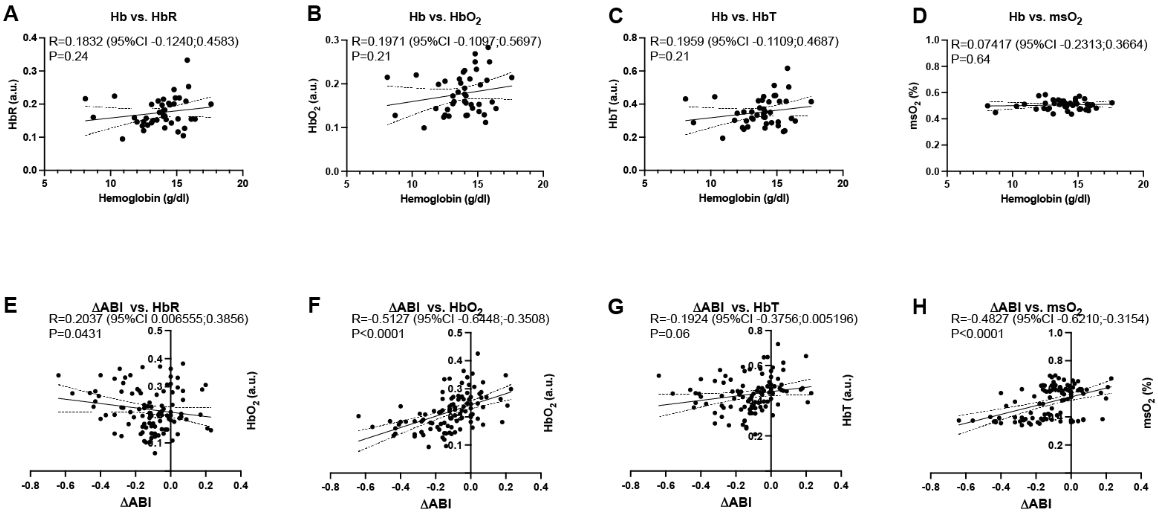

Supplementary Figure 2 – Correlation of blood hemoglobin level and MOST-derived hemoglobin parameters

R given as Pearson's correlation coefficient, Hb=blood hemoglobin level (mg/dl), HbR=MSOT-derived deoxygenated hemoglobin (a.u.), HbO_2=MSOT-derived oxygenated hemoglobin (a.u.), HbT=MSOT-derived total hemoglobin (a.u.), mSO_2=MSOT-derived oxygenation.

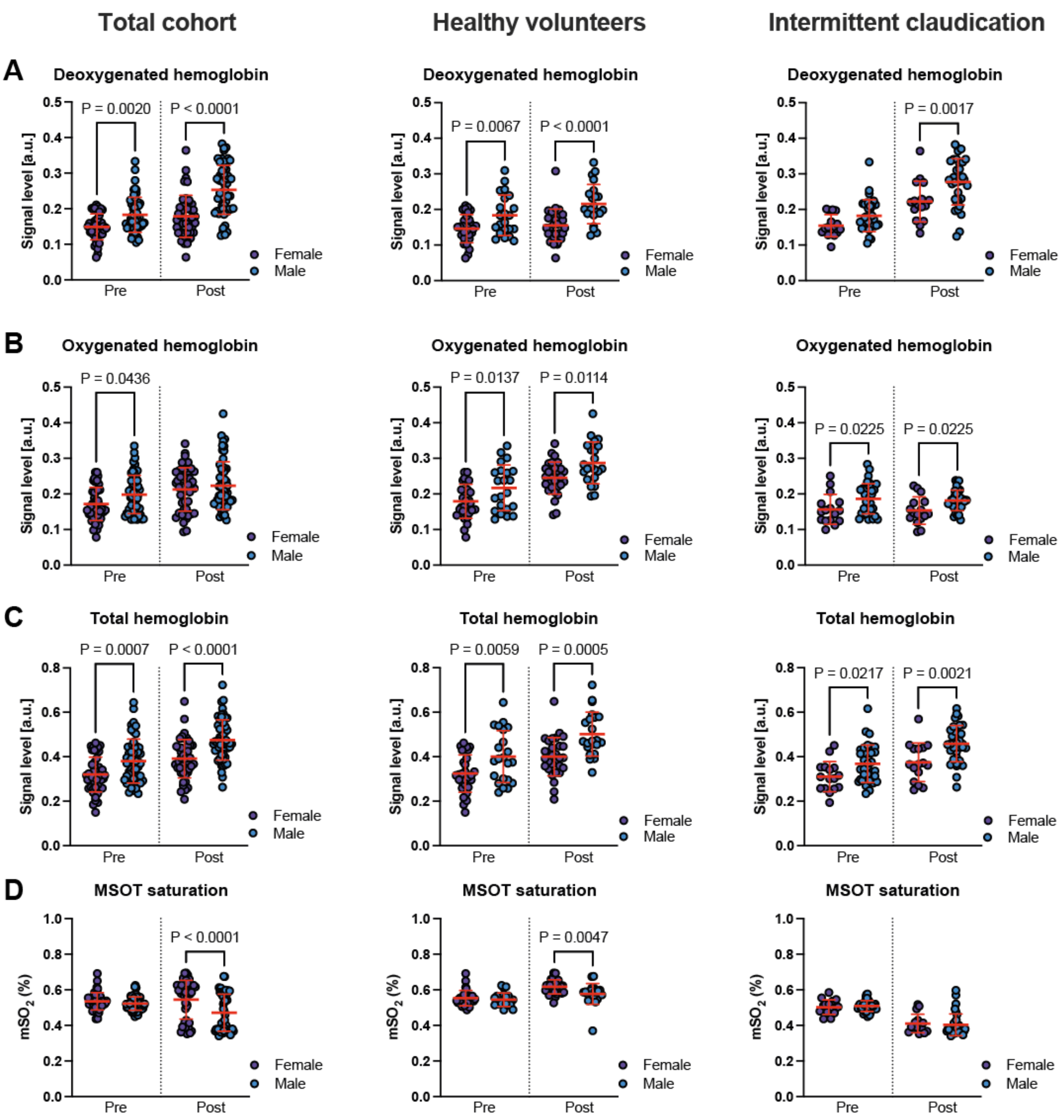

Supplementary Figure 3 – Gender differences in MSOT-derived parameters

(**A**) Deoxygenated hemoglobin levels before (Pre) and after exercise (Post) in the total cohort, healthy volunteers and patients with intermittent claudication. (**B**) Oxygenated hemoglobin levels before (Pre) and after exercise (Post) in the total cohort, healthy volunteers and patients with intermittent claudication. (**C**) Total hemoglobin levels before (Pre) and after exercise (Post) in the total cohort, healthy volunteers and patients with intermittent claudication. (**D**) MSOT-derived saturation (mSO_2) levels before (Pre) and after exercise (Post) in the total cohort, healthy volunteers and patients with intermittent claudication.

Supplementary Tables

	aTASC 1	aTASC 2	aTASC 3
**Interpretation**	angiographically healthy	good collateralization capability	poor collateralization capability
**Findings according to TASC II classification**	1. no findings in the AI area<br>2. no findings in FP area<br>3. no findings in IP area	1. no or A/B lesion in AI area<br>2. no or A/B/C/D lesion in FP area<br>3. any lesion in IP area<br>4. at least A/B lesions in 1. or 2.	1. C/D lesion in AI area<br>2. no or A/B/C/D lesion in FP area<br>3. any lesion in IP area

Supplementary Table 1 – aTASC classification based on the TASC II classification

TASC II, Trans-Atlantic Inter-Society Consensus Document on Management of Peripheral Arterial Disease classification II; aTASC, aggregated TASC II classification; AI, Aorto-iliac segment; FP, Femoral popliteal segment; IP, Infrapopliteal segment

Characteristic	Total cohort (n = 102)	HV (n = 52)		p-value	IC (n = 50)		p-value
		DC (n = 27)	VC (n = 25)		DC (n = 24)	VC (n = 26)	
**Age, yrs.**	63.83 ± 8.02	60.27 ± 6.66	59.64 ± 5.34	.712	68.21 ± 7.91	67.49 ± 8.07	.752
**Sex, n (%)**							
**Female**	46 (45.1)	18 (66.7)	12 (48.0)	.173	5 (20.8)	11 (42.3)	.104
**Risk factors, n (%)**							
**Smoking**	55 (53.9)	2 (7.4)	6 (24.0)	.098	24 (100.0)	23 (88.5)	.086
**Arterial hypertension**	56 (54.9)	6 (22.2)	5 (20.0)	.845	21 (87.5)	24 (92.3)	.571
**Dyslipidemia**	47 (46.1)	11 (40.7)	6 (24.0)	.199	14 (58.3)	16 (61.5)	.817
**Diabetes mellitus**	16 (15.7)	0 (0.0)	0 (0.0)	N/A	7 (29.2)	9 (34.6)	.680
**Obesity (BMI>30kg/m^2)**	12 (11.8)	1 (3.7)	0 (0.0)	.331	4 (16.7)	7 (26.9)	.382
**Positive family history**	45 (44.1)	10 (37.0)	13 (52.0)	.278	11 (45.8)	11 (42.3)	.802
**Relevant diseases, n (%)**							
**Coronary artery disease**	17 (16.7)	0 (0.0)	0 (0.0)	N/A	10 (41.7)	7 (26.9)	.272
**Carotid stenosis**	21 (20.6)	0 (0.0)	2 (8.0)	.134	9 (37.5)	10 (38.5)	.944
**History of myocardial infarction**	8 (7.8)	0 (0.0)	0 (0.0)	N/A	5 (20.8)	3 (11.5)	.370
**Stroke**	8 (7.8)	0 (0.0)	1 (4.0)	.294	5 (20.8)	2 (7.7)	.181
**Current medication, n (%)**							
**Antihypertensive**	54 (52.9)	6 (22.2)	5 (20.0)	.845	18 (75.0)	25 (96.2)	.031
**Lipid-lowering agent**	36 (35.3)	2 (7.4)	2 (8.0)	.936	15 (62.5)	17 (65.4)	.832
**Antidiabetic**	14 (13.7)	0 (0.0)	0 (0.0)	N/A	5 (20.8)	9 (34.6)	.278
**Previous revascularization procedure, n (%)**	22 (21.6)	0 (0.0)	0 (0.0)	N/A	8.8 (33.3)	14 (53.8)	.144
**Ankle-brachial-index**							
**Before the exercise**	0.84 ± 0.32	1.12 ± 0.06	1.11 ± 0.06	0.734	0.57 ± 0.26	0.54 ± 0.17	0.575
**After the exercise**	0.72 ± 0.42	1.07 ± 0.10	1.10 ± 0.10	0.308	0.35 ± 0.27	0.31 ± 0.16	0.522
**VASCUQOL-6 score**	18.65 ± 6.20	24.00 ± 0.00	24.00 ± 0.00	N/A	13.42 ± 2.95	12.77 ± 5.07	.581
**Walking distance in 6MWT [m]**							
**Relative**	115 ± 71	N/A	N/A	N/A	109 ± 55	120 ± 84	.594
**Absolute**	207 ± 88	N/A	N/A	N/A	200 ± 99	213 ± 80	.689
**Total**	463 ± 142	583 ± 34	578 ± 52	.649	342 ± 110	339 ± 88	.914
**PAD stage according to Fontaine, n (%)**							
**IIa**	24 (23.5)	0 (0.0)	0 (0.0)	N/A	15 (62.5)	9 (34.9)	.088
**IIb**	26 (25.5)	0 (0.0)	0 (0.0)	N/A	9 (37.5)	17 (65.4)	.088
**PAD stage according to aTASC, n (%)**							
**aTASC 1**	53 (52.0)	27 (100.0)	25 (100.0)	N/A	1 (4.2)	0 (0.0)	.480
**aTASC 2**	41 (40.2)	0 (0.0)	0 (0.0)	N/A	18 (75.0)	23 (88.5)	.281
**aTASC 3**	8 (7.8)	0 (0.0)	0 (0.0)	N/A	5 (20.8)	3 (11.5)	.305

Supplementary Table 2 – Demographic and Clinical Characteristics

Categorical data represented by absolute and relative frequencies, continuous data by mean ± standard derivation. Statistical comparison of the DC and VC using the Chi-squared test for categorical data and the t-test for continuous data. There is no significant difference between the two groups in the data considered (p-value <0.05). (HV, healthy volunteers; IC, patients with peripheral arterial disease with in Fontaine stage IIa/IIb or Rutherford category 1 to 3; DC, derivation cohort; VC, validation cohort; VASCUQOL-6, Vascular Quality of Life Questionnaire-6; 6MWT, Six-Minute-Walk-Test; PAD, peripheral arterial disease; aTASC (aggregated TASC II classification; Type 1: HV or IC with no signs of stenosis or occlusion in angiography; Type 2: signs of stenosis or occlusion in the femoropopliteal and/or infrapopliteal area or TASC-II-level A or B in the aortoiliac area; Type 3: TASC-II-level C or D in the aortoiliac area); N/A, not applicable)

	DC					VC	
	AUC	Sensitivity	Specificity	Youden index	Cut-off	Sensitivity	Specificity
**Pre**							
**Hb**	0.647 (0.495-0.799)	0.889 (0.719-0962)	0.250 (0.120-0.449)	0.14	0.211	0.154 (0.062-0.225)	0.880 (0.700-0.958)
**HbO_2**	0.582 (0.424-0.749	0.296 (0.159-0.485	0.917 (0.742-0.985)	0.21	0.232	0.885 (0.710-0.960)	0.400 (0.234-0.593)
**msO_2**	0.849 (0.741-0.956)	0.815 (0.633-0.918)	0.790 (0.595-0.908)	0.61	0.525	0.769 (0.580-0.890)	0.600 (0.407-0.766)
**Post**							
**Hb**	0.866 (0.766-0.965)	0.741 (0.553-0.868)	0.917 (0.742-0.985)	0.66	0.199	0.769 (0.580-0.890)	0.720 (0.524-0.857)
**HbO_2**	0.867 (0.764-0.971)	0.667 (0.478-0.814)	1.000 (0.862-1.000)	0.67	0.239	1.000 (0.871-1.000)	0.720 (0.524-0.857)
**msO_2**	0.991 (0.971-1.000)	1.000 (0.875-1.000)	0.958 (0.798-0.998)	0.96	0.523	0.962 (0.811-0.998)	0.960 (0.805-0.998)

Supplementary Table 3 – Representation and validation of the diagnostic quality of the MSOT measurement

The three MSOT parameters deoxygenated hemoglobin (Hb), oxygenated hemoglobin (HbO_2) and MSOT saturation (msO_2) are observed and analyzed before (Pre) and after (Post) the heel raise exercise. The area under the curve (AUC), sensitivity, specificity, and the cut-off point (calculated by using the optimal Youden index) are determined in the derivation cohort (DC). The cut-off point is applied in the validation cohort (VC) to validate the sensitivity and specificity calculated in the DC.

References

1. Nordanstig, J., et al., *Vascular Quality of Life Questionnaire-6 facilitates health-related quality of life assessment in peripheral arterial disease.* J Vasc Surg, 2014. **59**(3): p. 700-7.
2. Norgren, L., et al., *Inter-Society Consensus for the Management of Peripheral Arterial Disease (TASC II).* Eur J Vasc Endovasc Surg, 2007. **33 Suppl 1**: p. S1-75.
3. Antoniou, G.A., et al., *Commentary: TASC II Anatomic Classification for Infrapopliteal Arterial Disease: A Framework for Clinical Practice and Future Research.* J Endovasc Ther, 2015. **22**(5): p. 678-80.
4. Jaff, M.R., et al., *An update on methods for revascularization and expansion of the TASC lesion classification to include below-the-knee arteries: A supplement to the inter-society consensus for the management of peripheral arterial disease (TASC II): The TASC steering committee.* Catheter Cardiovasc Interv, 2015. **86**(4): p. 611-25.
