# Supplementary material for: Derivation and validation of a non-invasive optoacoustic imaging biomarker for detection of patients with intermittent claudication
Source: Commun Med (Lond). 2025 Mar 25;5:88. doi: 10.1038/s43856-025-00801-1 (PMC11937270; doi:10.1038/s43856-025-00801-1)
Supplement: Supplementary file 2 — Description of Additional Supplementary Materials [file 43856_2025_801_MOESM2_ESM.pdf]

Description of Additional Supplementary Files

File name: Supplementary Data 1

Description: the data underlying Fig. 2, Fig. 3 and Fig. 4
