# Supplementary material for: Derivation and validation of a non-invasive optoacoustic imaging biomarker for detection of patients with intermittent claudication
Source: Commun Med (Lond). 2025 Mar 25;5:88. doi: 10.1038/s43856-025-00801-1 (PMC11937270; doi:10.1038/s43856-025-00801-1)
Supplement: Supplementary file 4 — Reporting Summary [file 43856_2025_801_MOESM4_ESM.pdf]

nature portfolio

Corresponding author(s): Knieling, Ferdinand

Last updated by author(s): Feb 1, 2025

Reporting Summary

Nature Portfolio wishes to improve the reproducibility of the work that we publish. This form provides structure for consistency and transparency in reporting. For further information on Nature Portfolio policies, see our Editorial Policies and the Editorial Policy Checklist.

Statistics

For all statistical analyses, confirm that the following items are present in the figure legend, table legend, main text, or Methods section.

n/a	Confirmed	
☐	☒	The exact sample size (*n*) for each experimental group/condition, given as a discrete number and unit of measurement
☐	☒	A statement on whether measurements were taken from distinct samples or whether the same sample was measured repeatedly
☐	☒	The statistical test(s) used AND whether they are one- or two-sided<br>*Only common tests should be described solely by name; describe more complex techniques in the Methods section.*
☐	☒	A description of all covariates tested
☐	☒	A description of any assumptions or corrections, such as tests of normality and adjustment for multiple comparisons
☐	☒	A full description of the statistical parameters including central tendency (e.g. means) or other basic estimates (e.g. regression coefficient) AND variation (e.g. standard deviation) or associated estimates of uncertainty (e.g. confidence intervals)
☐	☒	For null hypothesis testing, the test statistic (e.g. *F*, *t*, *r*) with confidence intervals, effect sizes, degrees of freedom and *P* value noted<br>*Give P values as exact values whenever suitable.*
☒	☐	For Bayesian analysis, information on the choice of priors and Markov chain Monte Carlo settings
☒	☐	For hierarchical and complex designs, identification of the appropriate level for tests and full reporting of outcomes
☐	☒	Estimates of effect sizes (e.g. Cohen's *d*, Pearson's *r*), indicating how they were calculated

Our web collection on statistics for biologists contains articles on many of the points above.

Software and code

Policy information about availability of computer code

Data collection	n/a
Data analysis	n/a

For manuscripts utilizing custom algorithms or software that are central to the research but not yet described in published literature, software must be made available to editors and reviewers. We strongly encourage code deposition in a community repository (e.g. GitHub). See the Nature Portfolio guidelines for submitting code & software for further information.

Data

Policy information about availability of data

All manuscripts must include a data availability statement. This statement should provide the following information, where applicable:

- Accession codes, unique identifiers, or web links for publicly available datasets
- A description of any restrictions on data availability
- For clinical datasets or third party data, please ensure that the statement adheres to our policy

Deidentified data can be available after request by the authors.

Human research participants

Policy information about studies involving human research participants and Sex and Gender in Research.

Reporting on sex and gender	The data on sex was available from clinical records and confirmed by self-identification. A separate analysis was run for females and males to assess gender differences.
Population characteristics	HV had a mean age 63.8 ± 8.0 years and 45.1% were female. Patients with IC were 60.0 ± 6.0 years of age and 42.3% were female
Recruitment	The prospective data collection and analysis was designed as a monocentric, cross-sectional diagnostic study and performed at the Department of Vascular Surgery of the University Hospital Erlangen, Germany.
Ethics oversight	Ethics Committee of the University Erlangen-Nürnberg (22-62-Bm)

Note that full information on the approval of the study protocol must also be provided in the manuscript.

Field-specific reporting

Please select the one below that is the best fit for your research. If you are not sure, read the appropriate sections before making your selection.

☒ Life sciences ☐ Behavioural & social sciences ☐ Ecological, evolutionary & environmental sciences

For a reference copy of the document with all sections, see nature.com/documents/nr-reporting-summary-flat.pdf

Life sciences study design

All studies must disclose on these points even when the disclosure is negative.

Sample size	Statistical sample size calculation based on the results of the previous study (MSOT_PAD, NCT04641091) suggests a necessary study collective of at least 48 to a maximum of 124 subjects/patients. The two-sample t-test power calculation with a power of 0.8 gave an n of about 12 for each group (study group 1 - healthy volunteers, study group 1 – claudication patients, study group 2 - healthy volunteers, study group 2 - claudication patients). Additional sample size planning based on the AUC, which should be at least 0.7 for a relevant significance, gave an n of 31 subjects/patients for each group (with a significance level of 0.05 and a power of 0.8).<br>These calculations were based on data comparing healthy subjects with patients in stage IIb who, however, had not been adequately exercised (all only with the same walking distance of 150 meters). Due to the hypothesis of the study that the ability to differentiate between healthy subjects and claudication patients can be increased by adequate provocation testing, the sample size estimated here on the basis of the previous data is to be regarded as conservative. For this reason, after the inclusion of the first 25 patients and the first 25 healthy test persons,<br>the necessary total sample was reevaluated. During this interims analysis we found, a minimum sample size of 11 (oxygenated hemoglobin [HbO2])-33 (deoxygenated hemoglobin [Hb]) participants in each clinical group (HV, IC) (80% power, alpha of 0.05, two-sample t-test power calculation, two-sided).
Data exclusions	A total of 123 individuals were consecutively screened for enrollment between 9th May 2022 and 14th July 2022, of whom 115 gave written informed consent. In 109 cases, the study was completed with a full data set and finally 102 of them were used for data processing.
Replication	The repeatability of the method has been shown by Wagner AL: Precision of handheld multispectral optoacoustic tomography for muscle imaging, Photoacoustics, DOI: 10.1016/j.pacs.2020.100220 . Additionally, each MSOT measurement has been done over a time period of 20 seconds. Out of this time period, two different frames were chosen for analysis.
Randomization	Randomization was not possible in this trial, as patients were categorized according to their clinical stage.
Blinding	The examiners for the final MSOT data analysis were blinded to the results of the clinical assessments.

Reporting for specific materials, systems and methods

We require information from authors about some types of materials, experimental systems and methods used in many studies. Here, indicate whether each material, system or method listed is relevant to your study. If you are not sure if a list item applies to your research, read the appropriate section before selecting a response.

Materials & experimental systems

n/a	Involved in the study
☒	☐ Antibodies
☒	☐ Eukaryotic cell lines
☒	☐ Palaeontology and archaeology
☒	☐ Animals and other organisms
☐	☒ Clinical data
☒	☐ Dual use research of concern

Methods

n/a	Involved in the study
☒	☐ ChIP-seq
☒	☐ Flow cytometry
☒	☐ MRI-based neuroimaging

Clinical data

Policy information about clinical studies

All manuscripts should comply with the ICMJE guidelines for publication of clinical research and a completed CONSORT checklist must be included with all submissions.

Clinical trial registration

NCT05373927

Study protocol

https://classic.clinicaltrials.gov/ct2/show/NCT05373927

Data collection

Single center university hospital, study dates: 9th May 2022 and 14th July 2022

Outcomes

Study group 1: Derivation of optimal diagnostic thresholds for hemoglobin-associated MSOT parameters after a heel raise exercise in claudication patients and a healthy control group
Study group 2: Validation of the diagnostic accuracy of MSOT, employing the cut-off values derived in study group 1, regarding hemoglobin-associated MSOT parameters after a heel raise exercise in claudication patients and a healthy control group in an independent cohort

Primary endpoint:
Optimal diagnostic thresholds for hemoglobin-associated MSOT parameters before and after a heel raise exercise [Time Frame: single time point (1 day)]: optimal diagnostic thresholds for hemoglobin-associated MSOT parameters in calf muscle tissue in patients with intermittent claudication before and after a heel raise exercise

Details see: https://classic.clinicaltrials.gov/ct2/show/NCT05373927 (statistical analyses plan is provided online)
